# Hydroxychloroquine plus personal protective equipment versus standard personal protective equipment alone for the prevention of COVID-19 infections among frontline healthcare workers: the HydrOxychloroquine Prophylaxis Evaluation(HOPE) trial: A structured summary of a study protocol for a randomized controlled trial

**DOI:** 10.1186/s13063-020-04679-3

**Published:** 2020-08-31

**Authors:** Bharath Kumar Tirupakuzhi Vijayaraghavan, Vivekanand Jha, Dorrilyn Rajbhandari, Sheila Nainan Myatra, Oommen John, Arpita Ghosh, Abhinav Bassi, Sumaiya Arfin, Mallikarjuna Kunigari, Rohina Joshi, Lachlan Donaldson, Naomi Hammond, Balasubramanian Venkatesh, the HOPE investigators, Bharath Kumar Tirupakuzhi Vijayaraghavan, Vivekanand Jha, Balasubramanian Venkatesh, Vivekanand Jha, Sheila Nainan Myatra, Dorrilyn Rajbhandari, Oommen John, Arpita Ghosh, Abhinav Bassi, Sumaiya Arfin, Mallikarjuna Kunigari, Rohina Joshi, Lachlan Donaldson, Naomi Hammond, Santosh Kumar Nag, Syed Haider Mehdi Husaini, Viny Kantro, Ashita Singh, Arpit Mathew

**Affiliations:** 1 Department of Critical Care Medicine, Apollo Hospitals, Chennai, India; 2 The George Institute for Global Health, New Delhi, India; 3 School of Public Health, Imperial College, London, United Kingdom; 4 Manipal Academy of Higher Education, Manipal, India; 5 Academic Project Operations, Critical Care Program, The George Institute for Global Health, Sydney, New South Wales Australia; 6 Department of Anaesthesiology, Critical Care and Pain, Tata Memorial Hospital, Homi Bhabha National Institute, Mumbai, India; 7 Prasanna School of Public Health, MAHE, Manipal, India; 8 Faculty of Medicine, University of New South Wales, Sydney, Australia; 9 The George Institute for Global Health, Sydney, New South Wales Australia; 10 Royal North Shore Hospital, St Leonards, NSW Australia; 11 Wesley Hospital, Brisbane, Australia; 12 University of Queensland, Brisbane, Australia

**Keywords:** COVID-19, Randomised controlled trial, protocol, hydroxychloroquine, health personnel

## Abstract

**Objectives:**

To evaluate the effect of the combination of hydroxychloroquine (HCQ) and standard personal protective equipment (PPE) compared to the use of standard personal protective equipment alone on the proportion of laboratory confirmed COVID-19 infections among frontline healthcare workers(HCWs) in India

**Trial design:**

HOPE is an investigator initiated multi-centre open-label parallel group randomized controlled trial.

**Participants:**

All HCWs currently working in an environment with direct exposure to patients with confirmed COVID-19 infection are eligible to participate in the trial. The trial aims to be conducted across 20-30 centres (public and private hospitals) in India. HCWs who decline consent, who have a confirmed COVID-19 infection, those who are already on chloroquine/HCQ for any indication, or if pregnant or breast-feeding, or have known QT prolongation or are on medications that when taken with HCQ can prolong the QTc will be excluded.

**Intervention and comparator:**

The interventions to be compared in this trial are standard practice (use of recommended PPE) and HCQ plus standard practice. In the standard practice arm, HCWs will use recommended PPE as per institutional guidelines and based on their roles. They will be discouraged from taking HCQ to prevent contamination and contacted every week for the duration of the study to ascertain if they have taken any HCQ. Any such use will be reported as a protocol violation.

In the intervention arm, HCWs will be administered 800mg of HCQ as a loading dose on the day of randomization (as two 400mg doses 12hrs apart) and subsequently continued on 400mg once a week for 12 weeks. This will be in addition to the use of recommended PPE as per institutional guidelines and based on their roles. HCWs will collect the drug once every week from designated research and pharmacy staff at site. A weekly phone reminder will be provided to participants in this arm to ensure compliance. An ECG will be performed between 4-6 weeks in this arm and if the QTc is prolonged (greater than 450milliseconds), the drug will be stopped. Follow-up will however continue.

Participants in both arms will receive a weekly phone call for evaluation of the primary outcome, to monitor protocol compliance and development of any adverse events (in the HCQ group).

**Main outcomes:**

Participants will be followed on a weekly basis. The primary outcome is the proportion of HCWs developing laboratory confirmed COVID-19 infection within 6 months of randomization. We will also evaluate a number of secondary outcomes, including hospitalization related to suspected/confirmed COVID-19 infection, intensive care unit or high-dependency unit admission due to suspected/confirmed COVID-19 infection, all-cause mortality, need for organ support ( non-invasive or invasive ventilation, vasopressors and renal replacement therapy), ICU and hospital length of stay, readmission, days off work and treatment-related adverse events.

**Randomisation:**

Randomisation will be conducted through a password-protected, secure website using a central, computer-based randomisation program. Randomisation will be stratified by participating institutions and by the role of HCW – nursing, medical and other. Participants will be randomised 1:1 to either standard practice only or HCQ plus standard practice. Allocation concealment is maintained by central web-based randomisation

**Blinding (masking):**

This is an unblinded study: study assigned treatment will be known to the research team and participant. Bias will be mitigated through an objective end point (laboratory confirmed COVID-19 infection).

**Numbers to be randomised (sample size):**

A total of 6,950 HCWs will be enrolled (3475 to the intervention) and (3475 to the standard practice group) to detect a 25% relative reduction, or 2.5% absolute reduction, in the infection rate from an estimated baseline infection rate of 10%, with 80% statistical power using a two-sided test at 5% level of significance. Available data from China and Italy indicate that the rate of infection among frontline healthcare workers varies between 4% to 12%. We therefore assumed a baseline infection rate of 10% among HCWs. This sample size allows for a potential loss to follow-up rate of 10% and a potential non-compliance rate of 10% in both the treatment and control arms.

**Trial Status:**

HOPE protocol version 3.0 dated June 3^rd^ 2020. Recruitment started on 29^th^ June 2020 and currently 56 participants have been enrolled. Planned completion of enrolment is January 31^st^ 2021.

**Trial registration:**

Clinical Trials Registry of India: CTRI/2020/05/025067 (prospectively registered) Date of registration: 6^th^ May 2020

**Full protocol:**

The full protocol is attached as an additional file, accessible from the Trials website (Additional file 1). In the interest of expedited dissemination of this material, the familiar formatting has been eliminated; this Letter serves as a summary of the key elements of the full protocol.

The study protocol has been reported in accordance with the Standard Protocol Items: Recommendations for Clinical Interventional Trials (SPIRIT) guidelines (Additional file 2).

## Supplementary information

**Additional file 1.**

**Additional file 2.**

## Data Availability

Not applicable.

