# Supplementary material for: Hydroxychloroquine plus personal protective equipment versus standard personal protective equipment alone for the prevention of COVID-19 infections among frontline healthcare workers: the HydrOxychloroquine Prophylaxis Evaluation(HOPE) trial: A structured summary of a study protocol for a randomized controlled trial
Source: Trials. 2020 Aug 31;21:754. doi: 10.1186/s13063-020-04679-3 (PMC7456761; doi:10.1186/s13063-020-04679-3)
Supplement: Supplementary file 1 — Additional file 1. [file 13063_2020_4679_MOESM1_ESM.pdf]

HOPE HCQ 4 Healthcare Workers

HOPE - **H**ydr**O**xychloroquine **P**rophylaxis **E**valuation

HOPE Study

A randomized controlled trial of hydroxychloroquine (HCQ) compared to standard practice for the prevention of COVID-19 infections among healthcare workers (HCW) exposed to SARS-CoV2

Protocol Number: TGI-IN4673

Version Number: 3.0

Date: 03 June 2020

The George Institute for Global Health

Confidentiality statement: This entire protocol is the intellectual property of the investigators and cannot be used without express written permission.

Contents

1. Study Synopsis 4
2. Administrative information....................5
2.1. Chief Investigators 5
2.2. Study Sponsor / Trial Coordinating Centre 5
2.3. HOPE Management Committee....................5
2.4. Trial Funding 5
2.5. Role of the funding bodies....................5
2.6. Trial Registration....................5
3. Introduction 6
3.1. Background 6
3.2. Rationale 7
4. Study Design 8
4.1 Aim 8
4.2 Hypothesis....................8
4.3 Design....................8
5. Study Outcomes....................8
5.1 Primary Outcome 8
5.2 Secondary Outcome....................8
6. Study Participants 8
6.1. Study Setting 8
6.2. Inclusion criteria....................8
6.3. Exclusion criteria: 9
7. Study interventions....................9
7.1. Participant recruitment....................9
7.2. Randomisation 9
7.3. Study treatment regimen....................9
7.3.1 Standard practice (use of PPE only) (control arm)....................9
7.3.2. HCQ and standard practice (use of PPE) (intervention arm) 10
7.4. Premature cessation of study assigned treatment....................10
7.5. Blinding 10
7.6. Safety considerations....................10
7.6.1. Management of potential risks to participants 10
7.6.2. Precautions and adverse reactions....................10
8. Study Assessments....................11
8.1. Participating site information 11
8.2. Screening....................11
8.3. Randomisation 11

8.4. Baseline 11
8.5. Weekly questionnaire 11
8.6. Electrocardiograph 11
8.7. Definition of COVID-19 diagnosis 11
8.8. COVID-19 and Follow up at 6 months. 11
9. Safety Monitoring and reporting 11
9.1. Safety Reporting: 11
9.1.1. Definition of Serious Adverse Events: 11
9.1.2. Causality 13
9.1.3. Procedures for Recording Adverse Events 13
9.1.4. Reporting 13
9.2. Data Safety Monitoring Committee 13
10. Ethics and dissemination 14
10.1. Ethical principles 14
10.2. Human Research Ethics Committee 14
10.3. Informed consent procedures 14
10.4. Confidentiality and privacy 14
11. Data collection and management 14
11.1. Record retention 15
12. Quality control and quality assurance monitoring 15
12.1. Responsibilities of the investigator 15
12.2. Responsibilities of the Coordinating centre 15
12.2.1. Site Initiation 15
12.2.2 Monitoring during the study 16
12.2.3. Site Close out 16
12.3. Management of protocol deviations 16
12.4. Access to data and documents 16
13. Statistical methods 17
13.1. Sample size: 17
13.2. Statistical analysis plan 17
13.3. Interim analysis 17
14. Publications and reports 17
14.1. Public Access 17
15. References: 18

1. Study Synopsis

**Study Title**	A randomized controlled trial of hydroxychloroquine (HCQ) compared to standard practice for the prevention of COVID-19 infections among healthcare workers (HCW) exposed to SARS-CoV2
**Short Title**	HOPE - **H**ydr**O**xychloroquine **P**rophylaxis **E**valuation.
**Protocol Number**	Version 3.0, 03 June 2020
**Study Design**	Multi-centre, open, phase III, randomized controlled trial of standard practice vs. standard practice plus HCQ
**Primary outcome**	Proportion of laboratory confirmed COVID-19 infections at 6 months after randomisation
**Secondary outcomes**	1. Hospitalization due to suspected COVID-19 infection<br>2. admission with suspected or confirmed COVID-19 to a HDU or ICU<br>3. all-cause mortality<br>4. need for ventilation (O2 therapy, non-invasive or invasive)<br>5. need for vasopressors<br>6. need for renal replacement therapy<br>7. duration of hospitalization<br>8. duration of ICU or HDU stay<br>9. readmission to hospital<br>10.days off work
**Intervention**	HCW will be randomised to either receive 400mg of HCQ twice on the day of enrollment followed by 400mg once a week plus standard practice (use of PPE) or standard practice (use of PPE) for 12 weeks and followed up at 6 months
**Planned sample size**	6,950 HCWs
**Planned study period**	12 months
**Inclusion criteria**	Health Care Worker (medical, nursing, allied health, ancillary worker, visiting doctor) currently working in an environment with direct exposure to patients with confirmed COVID-19 infection
**Exclusion criteria**	1. HCW refused/did not grant consent<br>2. HCW has a laboratory confirmed of COVID-19 infection<br>3. HCW is currently taking chloroquine or HCQ<br>4. HCW is pregnant<br>5. HCW is breast feeding<br>6. HCW has known QT prolongation<br>7. HCW is currently taking any of the medications that are contra indicated in combination with HCQ:<br>• Antiarrythmics (Amiodarone),<br>• Systemic Antimicrobials (Azithromycin, Fluconazole, Itraconazole, Ketoconazole, Ciproflxacin, Ofloxacin, Levofloxacin, Efavirenz),<br>• Antipsychotics/ Antidepressants (Olanzapine, Fluoxetine)<br>• Prokinetics/antimetics/H2 blockers (Cisapride, Domeridone, Famotidine),<br>• Cardiac medications (Ranolazine, Ivabradine)<br>8. HCW has history of serious cardiac dysrhythmias or cardiomyopathy<br>9. HCW has maculopathy of the eye (a contra-indication to HCQ)<br>10. HCW is immunocompromised due to a disease or therapy
**Randomisation**	Web-based randomisation system available 24/7. Eligible HCWs will be randomized in a 1:1 ratio to standard practice plus HCQ or standard practice only.

2. Administrative information

2.1. Chief Investigators

Prof. Vivekanand Jha, Director, The George Institute for Global Health, New Delhi, India

Dr. Bharath Kumar Tirupakuzhi Vijayaraghavan, Consultant, Critical Care, Apollo Hospitals, Chennai, India

2.2.Study Sponsor / Trial Coordinating Centre

Sponsor: The George Institute for Global Health, New Delhi, India

2.3.HOPE Management Committee

Indian investigators:

Dr. Arpita Ghosh, Senior Research Fellow, The George Institute for Global Health, New Delhi, India

Dr. Oommen John, Senior Research Fellow, The George Institute for Global Health, New Delhi, India

Prof. Bala Venkatesh, Professorial Fellow, The George Institute for Global Health, Australia and Adjunct Prof. St.Johns Medical College and Research Institute, Bangalore, India

Prof. Sheila Myatra, Anaesthesiology and Intensive Care, Tata Memorial Hospital and Research Institute, Mumbai, India

Dr Rohina Joshi, The George Institute for Global Health, India and Australia

International co-investigators:

Dr Naomi Hammond, Senior Research Fellow, The George Institute for Global Health, Australia

Dr Lachlan Donaldson, Research Fellow, The George Institute for Global Health, Australia

Dorrilyn Rajbhandari, Senior Project Manager, The George Institute for Global Health, Australia

2.4. Trial Funding

The George Institute for Global Health has received support from Wesley Medical Research, Australia and is the trial funding body for sites in India. Additional funding will be sought.

2.5.Role of the funding bodies

The study will be designed and conducted, and the results analysed, presented and published by the investigators independent of any funding agencies.

2.6. Trial Registration

The protocol has been registered on the following clinical trial registry: CTRI/2020/05/025067

3. Introduction

3.1. Background

In December 2019, a novel strain of the coronavirus (now labelled SARS CoV-2) emerged from Wuhan (Hubei province), China and resulted in a cluster of cases of respiratory illness with several patients proceeding onto severe acute respiratory failure. The disease which has now been labelled COVID-19 has rapidly spread and as of 27th March 2020, involves 200 countries (or territories) with 1812734 cases and 113675 deaths globally.[1] The World Health Organization (WHO) declared the disease a Pandemic on the 11th of March, 2020.[2]

In India, after an initial period of isolated reports (mostly imported cases), there has been a steep increase in the number of cases to over 35043 by the 1st of May with 1147deaths.[3].[3] From initial reports, it appears that about 15-20% of affected patients need hospitalization and/or intensive care[4,5] with conflicting reports of the case fatality rate (anywhere between 0.15-15.8%).[6]

Healthcare workers (HCWs) who are at the frontlines of the battle against COVID-19 are at very high risk of acquiring infection. In published data from China[4], 3.8% of confirmed cases were healthcare workers and in Italy about 9%[7]. In the previous Severe Acute Respiratory Syndrome (SARS) and Middle East Respiratory Syndrome (MERS) coronavirus infections, HCWs accounted for 21.1% and 19.1% of cases respectively.[8,9] Based on epidemiological modelling from the data reported from China[5] with 10-15% of patients requiring hospitalization and 5% requiring intensive care, this could translate into hundreds of thousands of patients needing hospital care in India. This will potentially put thousands of HCWs at risk.

Whilst equipment capacity and health care systems can be boosted during pandemics, HCWs cannot be urgently manufactured. Presently, health care workers are every country's most valuable resource. **There is no proven effective prophylaxis, treatment or vaccine.** The availability of personal protective equipment (PPE) for HCWs, a key factor for HCW safety, is also a concern as shortages have been described in many regions protecting the HCWs is likely to be particularly challenging because of the limited isolation facilities, limited personal protective equipment (PPE), and higher patient density per ward. This poses the problems of increasing the case burden, loss of trained healthcare personnel that are vital for patient care and additionally the massive loss of morale among the entire healthcare community. There are reports that there is severe shortage of PPEs,[10] which makes it imperative to find alternate strategies that provide protection against this infection.

Given the large population and the relatively low testing rates, there will continue to be a pool of infected community members whom will contribute to ongoing new infections even after the peak of the pandemic is over. There are now reports confirming that over 10% of patients admitted with severe respiratory illness to hospitals had no travel history. [11]

Although several groups around the world are racing to develop a vaccine, most estimates suggest it will take over a year to develop one. Currently, there are no proven therapies for COVID-19.[12] There are in-vitro data that confirm efficient inhibition of SARS-CoV2 by both Chloroquine and Hydroxychloroquine (HCQ) [13,14] and several trials are now underway for investigating the effectiveness of these drugs in patients with confirmed COVID-19. We note that the WHO has started a mega-trial of four different treatment approaches, but only for treatment of patients with COVID-19 infection.[15] Both drugs have been in use for several years (for malaria and for rheumatoid arthritis respectively) and have a proven track record of safety.

Recently, the Indian Council of Medical Research (ICMR) has recommended the use of HCQ for prophylaxis of HCW, but have made a plea that "proof of concept and pharmacokinetics studies be taken up expeditiously" and said that "new evidence will guide any change in the recommendation". UK has prohibited its use outside of clinical trials. Furthermore, there have been recent reports of adverse events [16]including death following the use of HCQ as prophylaxis/treatment for this condition. There is

an ethical and a public health imperative to generate definitive clinical trial evidence for the efficacy and safety of this approach in HCW.

To answer this question, we propose a randomized controlled trial of HCQ among healthcare workers exposed to confirmed COVID-19 patients. We hypothesise that the use of HCQ as a prophylactic agent amongst frontline healthcare workers will reduce the incidence of new COVID-19 infections. The primary outcome of interest will be the proportion HCWs that develop a confirmed COVID-19 infection, while being on prophylaxis. We also propose to align our study with similar studies being led globally to ensure a coordinated meta-analysis.

3.2. Rationale

Healthcare workers involved in the care of hospitalized COVID-19 patients are at high risk, even with the conscientious use of personal protective equipment. These risks are further amplified in those taking care of patients in the intensive care unit (ICU), where procedures that are part of patient's care (such as nebulizations, intubation, non-invasive ventilation, cardio-pulmonary resuscitation etc.) dramatically increase the risk of aerosol generation and the potential for HCW infection. The loss of a part of or significant portions of the healthcare workforce will seriously impede efforts at controlling the pandemic and will likely result in collapse of the entire healthcare system.

There is biological plausibility that hydrocychloroquine (HCQ) will provide effective prophylaxis against SARS-CoV-2 infection based on its ability to reduce binding of the virus to the ACE2 receptor, prevent cellular entry of the virus and inhibit viral replication. HCQ has many favourable pharmacokinetic characteristics such has high oral bioavailability, a very large volume of distribution, a long terminal half-life, and is concentrated in tissues including the lung Chloroquine and hydroxychloroquine have both shown in-vitro effect against the virus (both in the previous SARS epidemic and now for SARS-CoV2). Both drugs have been in use for a several years and extended duration use has shown to be safe. Chloroquine continues to be used as chemoprophylaxis by travellers visiting regions where malaria is endemic and hydroxychloroquine is part of the standard therapy for conditions such as SLE and Rheumatoid Arthritis. Both drugs are inexpensive and relatively easily available across the country.

<u>Rationale for HCQ prophylaxis dosage and duration</u>

There are no human studies of HCQ prophylaxis against COVID-19. However, there is an advisory recommendation from the Indian Council of Medical Research for HCW to take a loading dose of 400mg taken twice on Day 1 followed by 400mg once a week for 7 weeks. Based on the current number of cases in India and the International trajectories of COVID -19 cases we anticipate HCW will be exposed for longer than 7 weeks. Therefore a 12-week prophylaxis duration has been chosen for this study. There is reasonable safety data on use of HCQ for longer than 12 weeks and therefore is a safe duration for this trial.

Only a robust, well designed and implemented RCT would provide reliable estimates of benefit and provide data on adverse events (AEs) and serious adverse events (SAEs).

4. Study Design

4.1 Aim

To conduct a multicentre randomised, controlled trial (RCT) to determine whether hydroxychloroquine (HCQ) in addition to standard practice reduces the proportion of HCWs developing symptomatic and laboratory confirmed COVID-19 infections as compared to standard practice alone.

4.2 Hypothesis

The HOPE study will test the hypothesis that a weekly dose of 400mg of oral HCQ for 12 weeks (following a HCQ loading dose, 400mg twice on the day of enrolment) in addition to standard practice (using PPE) is superior to standard practice (using PPE) alone at reducing the risk of acquiring laboratory-confirmed COVID-19 infection in frontline healthcare professionals caring for or in contact with patients with known or suspected COVID-19 disease.

4.3 Design

This is a, multi-centre, open, phase III, randomized controlled trial of HCQ prophylaxis+ standard practice vs. standard practice alone.

5. Study Outcomes

5.1 Primary Outcome

The proportion of laboratory confirmed COVID-19 cases within 6 months of randomisation

5.2 Secondary Outcome:

1) hospitalization due to suspected COVID-19 disease
2) ICU or HDU admission due to suspected COVID-19
3) all-cause mortality
4) need for mechanical ventilation (O2 therapy, non-invasive or invasive)
5) need for vasopressors
6) need for renal replacement therapy
7) hospital length of stay
8) ICU or HDU length of stay
9) readmission to hospital
10) days absent from work due to suspected or confirmed COVID-19

6. Study Participants

6.1. Study Setting

This study will be conducted in public and private hospitals across India. These centres will be selected on the basis of them being designated centres for COVID-19 patients by the Government of India or involved in the care of patients with confirmed COVID-19 infection.

6.2. Inclusion criteria

1. Health Care Worker (medical, nursing, allied health, ancillary worker, visiting doctor) currently working in an environment with direct exposure to patients with confirmed COVID-19 infection.

6.3. Exclusion criteria:

1. HCW refused/did not grant consent
2. HCW has a laboratory confirmed COVID-19 infection
3. HCW is currently taking chloroquine or HCQ
4. HCW is pregnant
5. HCW is currently breast feeding
6. HCW has a known history of QT prolongation
7. HCW is currently taking any of the medications that are contra indicated in combination with HCQ:
    - Anti-arrhythmic (Amiodarone),
    - Systemic Antimicrobials (Azithromycin, Fluconazole, Itraconazole, Ketoconazole, Ciproflxacin, Ofloxacin, Levofloxacin, Efavirenz),
    - Antipsychotics/ Antidepressants (Olanzapine, Fluoxetine),
    - Prokinetics/antimetics/H2 blockers (Cisapride, Domeridone, Famotidine),
    - Cardiac medications (Ranolazine, Ivabradine)
8. HCW has a history of serious cardiac dysrhythmias or cardiomyopathy
9. HCW has maculopathy of the eye (contra-indicated in the use of HCQ)
10. HCW is immunocompromised due to a disease or therapy

7. Study interventions

7.1.Participant recruitment

Potential participants will be identified by research staff in each of the sites and will be approached to consent to participate in the study. The recruitment period is expected to be 6 months.

7.2. Randomisation

Randomisation will be conducted through a password-protected, secure website using a central, computer-based randomisation program. Randomisation will be stratified by participating institution and by the role of HCW – nursing, medical and other. Participants will be randomised 1:1 to either standard practice only or HCQ plus standard practice.

Following successful randomisation, each participant will be assigned a unique ‘participant study number’.

7.3. Study treatment regimen

7.3.1 Standard practice (use of PPE only) (control arm)

Standard practice will be defined as the use of personal protective equipment (PPE) as per the recommendations of the institution employing the HCW. The recommendation of the participating institutions will be ascertained by obtaining any policies or guidelines relating to the use of PPE when caring for or exposed to COVID-19 patients. This definition is pragmatic in nature, due to the context of an ongoing pandemic at the time of the trial and given that time is of the essence.

HCWs randomised to ‘standard practice’ will continue to use PPE whilst at work as per their institutional recommendations. They will be discouraged from taking HCQ. Participant will be asked weekly if they have taken HCQ and this will be reported as a protocol deviation.

7.3.2. HCQ and standard practice (use of PPE) (intervention arm)

HCWs will receive 400mg of HCQ twice on the day of enrollment, followed by 400mg once a week for a total of 12 weeks. The HCQ on the day of enrolment will be given as two doses of 400mg, one dose under supervision by the research staff at site, the other dose will be given to the HCW to take later. Study drug (HCQ) will be provided on a weekly basis for 12 weeks by the site study team and will require the HCW to attend to receive the weekly dose.

All HCW in the HCQ plus standard practice arm will be required to have an ECG performed once between weeks 4 to 6. An ECG should be performed if the HCW reports experiencing side effects such as chest pain, syncope and/or palpitations.

HCWs randomized to 'HCQ plus standard practice' will continue to use PPE whilst at work as per their institutional recommendations, however they will receive HCQ weekly as prophylaxis against contracting COVID-19 infection.

7.4. Premature cessation of study assigned treatment

For HCWs assigned to the HCQ plus standard practice arm, study drug will be suspended if the HCW contracts COVID-19 during the 12 weeks treatment period.

Study drug may also be permanently stopped in the following circumstances:

1. Request to stop the study drug by the participant. Consent to collect follow-up data will be sought.
2. Adverse or serious adverse reaction to HCQ.

Regardless of whether the full study treatment regime is continued or not, the follow-up schedule should continue unchanged for all randomised participants.

7.5. Blinding

This is an unblinded study: study assigned treatment will be known to the research team and participant. Bias will be mitigated through an objective end point (laboratory confirmed COVID-19 infection).

7.6. Safety considerations

7.6.1. Management of potential risks to participants

HCQ has a favorable adverse effect profile and has been in use for Rheumatoid arthritis and systemic lupus erythematosus for several years. Most adverse effects are minor and usually restricted to nausea, stomach cramps, headache and diarrhoea. However, all AEs and SAEs will be recorded and reported to the Ethics Committee, Sponsor and the required regulatory bodies.

In accordance with the Indian Council of Medical Research recommendations and as a safety strategy, an ECG will be performed once between weeks 4 to 6 weeks for all HCWs randomized to receive HCQ. An ECG will be performed if the HCW reports cardiovascular symptoms such as chest pain, syncope and/or palpitations. If the ECG report indicates QT prolongation (QT Interval is >45ms) and /or is abnormal the HCQ will be ceased and the participant referred to a cardiologist.

7.6.2. Precautions and adverse reactions

HCQ is a registered product with the Central Drugs Standard Control Organization (CDSCO). The researchers must be aware of the precautions and potential adverse reactions for HCQ that are detailed in Product Information for India. Participants will be monitored for the known side effects of HCQ including ECG monitoring for abnormal prolongation of the QT interval (QT Interval >450ms).

8. Study Assessments

8.1. Participating site information

Data describing institutional recommendations for HCWs on the use of PPE when caring for or exposed to COIVD-19 patients will be collected prior to recruitment commencing at all sites.

8.2.Screening

Potential participants will be approached by the research team who will screen them for eligibility and request consent.

A screening log will be kept to monitor recruitment.

8.3. Randomisation

Once consent is obtained, the participants demographics will be entered into a web-based randomisation system. Each eligibility criterion will be answered with a Yes / No response and only participants meeting all criteria will proceed to randomisation.

8.4.Baseline

At baseline, information will be collected on designation, role in COVID ward (nursing, medical, allied health, ancillary worker, visiting doctor), demographics, average shift duration, and comorbidities.

8.5.Weekly questionnaire

Each participant will complete a questionnaire either by phone interview or in person. Information will be obtained about the amount of exposure during the past week, confirmation of compliance with standard process and HCQ administration or not. This will continue for a total of 25 weeks unless the participant tests positive to COVID-19.

8.6.Electrocardiograph

Each participant in the HCQ plus standard practice arm will have an ECG performed once between weeks 4 to 6 following randomisation. The ECG will be assessed for QT interval prolongation (QT interval is >450ms) and HCQ will be ceased if the report is abnormal. An ECG may performed if the HCW reports cardiovascular symptoms such as chest pain, syncope and/or palpitations.

8.7.Definition of COVID-19 diagnosis

COVID-19 diagnosis is defined as a positive laboratory confirmed COVID-19 infection.

8.8. COVID-19 and Follow up at 6 months.

When a participant test indicates COVID-19 positive then administration of HCQ ceases and the information is collected at the end of 6 months on hospitalisation, admission to HDU or ICU, the need for ventilation (O2 therapy, non-invasive and invasive), Inotropes and renal replacement therapy and the amount of time off work.

9. Safety Monitoring and reporting

9.1. Safety Reporting:

9.1.1. Definition of Serious Adverse Events:

A serious adverse event is any untoward medical occurrence that:

- results in death
- is life-threatening
- requires inpatient hospitalisation or prolongation of existing hospitalisation
- results in persistent or significant disability/ incapacity
- consists of a congenital anomaly or birth defect

Other ‘important medical events’ may also be considered serious if they jeopardise the participant or require an intervention to prevent one of the above consequences.

Definitions:

Adverse Event (AE)	Any untoward medical occurrence in a participant to whom a medicinal product has been administered, including occurrences which are not necessarily caused by or related to that product.
Adverse Reaction (AR)	An untoward and unintended response in a participant to an investigational medicinal product which is related to any dose administered to that participant.<br>The phrase "response to an investigational medicinal product" means that a causal relationship between a trial medication and an AE is at least a reasonable possibility, i.e. the relationship cannot be ruled out i.e. the relationship is definitely, probably, possibly or unlikely to be related (see below).<br>All cases judged by either the reporting medically qualified professional or the Sponsor as having a reasonable suspected causal relationship to the trial medication qualify as adverse reactions.
Serious Adverse Event (SAE)	A serious adverse event is any untoward medical occurrence that:<br>• results in death<br>• is life-threatening<br>• requires inpatient hospitalisation or prolongation of existing hospitalisation<br>• results in persistent or significant disability/incapacity<br>• consists of a congenital anomaly or birth defect.<br>Other ‘important medical events’ may also be considered serious if they jeopardise the participant or require an intervention to prevent one of the above consequences.<br>NOTE: The term "life-threatening" in the definition of "serious" refers to an event in which the participant was at risk of death at the time of the event; it does not refer to an event which hypothetically might have caused death if it were more severe.
Serious Adverse Reaction (SAR)	This is an adverse event that is both serious and is considered a drug reaction.
Suspected Unexpected Serious Adverse Reaction (SUSAR)	A SUSAR is a SAR that is:<br>• not listed in the summary of product characteristics (SmPC) for that product or

	• has not been described in the published literature before
Expectedness	An expected AR or SAR is a drug reaction that is listed in the SmPC and or has been described in the published literature before

NB: to avoid confusion or misunderstanding of the difference between the terms "serious" and "severe", the following note of clarification is provided: "Severe" is often used to describe intensity of a specific event, which may be of relatively minor medical significance. "Seriousness" is the regulatory definition supplied above.

9.1.2. Causality

The relationship of each adverse event to the trial medication must be determined by a medically qualified individual according to the following definitions:

- Definitely related
- Probably related
- Possibly related
- Unlikely to be related
- Not related

9.1.3. Procedures for Recording Adverse Events

All AEs occurring during the trial/ or until 28 days after the trial finishes, that are observed by the Investigator or reported by the participant, will be recorded on the CRF, whether or not attributed to trial medication.

The following information will be recorded: description, date of onset and end date, severity, assessment of relatedness to trial medication, other suspect drug or device and action taken. Follow-up information should be provided as necessary.

The severity of events will be assessed following the Common Toxicity Criteria v5.0: 1 = mild, 2 = moderate, 3 = severe, 4 = very severe.

AEs considered related to the trial medication as judged by a medically qualified investigator or the Sponsor will be followed either until resolution, or the event is considered stable.

It will be left to the Investigator's clinical judgment to decide whether or not an AE is of sufficient severity to require the participant's removal from treatment. A participant may also voluntarily withdraw from treatment due to what he or she perceives as an intolerable AE. If either of these occurs, the participant must undergo an end of trial assessment and be given appropriate care under medical supervision until symptoms cease, or the condition becomes stable.

9.1.4. Reporting

All SAEs, including SARs and SUSARs, detected by site investigator should be reported to the co-principal investigators and the local ethics committee within 24hrs.

The co-principal investigators will also report these to the Sponsor within 24hrs of becoming aware of the event.

9.2. Data Safety Monitoring Committee

An independent Data Safety and Monitoring Committee with a fully constituted DSMC charter will be formed to oversee the progress of the trial and to conduct interim analyses. In order to address safety concerns, at least one formal interim analysis will be conducted after 90 days of enrolment. The purpose of this interim analysis is to test for the difference in outcomes between the two study groups, to check for potential safety issues as well as assess early efficacy. Any additional reviews of the data or may be performed at the discretion of the Independent Data Monitoring Committee.

10. Ethics and dissemination

10.1. Ethical principles

The study will be conducted in accordance with ethical principles consistent with the Declaration of Helsinki[19] and all relevant national and local guidelines on the ethical conduct of research.[20-21]

10.2. Human Research Ethics Committee

Ethics Committee Approval:
All participating sites and investigators will obtain local EC approval and other approvals as necessary.

10.3. Informed consent procedures

Consent:

All eligible participants will be approached for participation in the trial by a member of the site research team.

To obtain informed consent, study personnel will follow the following steps:

- Present information on the study in a simple and understandable manner;
- Answer questions in a simple and understandable manner;
- Allow the potential participant an opportunity to reflect and discuss study participation with their family, friends, or family physician if desired;
- Confirm that the participant understands the risks and benefits of participating in the study and that their participation is voluntary;
- Complete and obtain signatures for informed consent form and obtain contact information from the participant.

10.4. Confidentiality and privacy

All participant information pertaining to the study will be stored in a computer database maintaining confidentiality in accordance with local legislation regarding privacy and use of health data. When archiving or processing data pertaining to the investigator and/or to study participants, the coordinating centre will take all appropriate measures to safeguard and prevent access to this data by any unauthorised third party.

The site Principal Investigator will maintain the confidentiality of all study documentation and take measures to prevent accidental or premature destruction of these documents. The site Principal Investigator must notify the coordinating centre prior to destroying any study documents following study completion or discontinuation. If the site Principal Investigator's situation is such that archiving can no longer be ensured by him/her, the site Principal Investigator will inform the coordinating centre and the relevant records will be transferred to a mutually agreed designee.

If any site Principal Investigator retires, relocates, or otherwise withdraws from conducting the study, the responsibility for maintaining records may be transferred to the coordinating centre, or other site Principal Investigator. The coordinating centre must be notified of and agree to the change. All associated documentation must also be updated.

11. Data collection and management

Data management will be provided by The George Institute for Global Health, India. The principle means of data collection and data processing will be electronic via a password protected website (electronic Case Report Form - eCRF). All computerised forms will be electronically signed by the authorised study

staff and all changes made following the electronic signing will have an electronic audit trail with a signature and date.

The relevant study participant data will be collected on paper during interviews with the participants by the site research staff, either in person or by telephone call. Participants will be contacted via telephone weekly for 25 weeks after randomisation by a member of the research team at each site to determine treatment compliance and COVID-19 status. Any paper CRFs and participants logs will be kept at the participating sites in secure locked cabinets or other enclosures that are accessible only to study personnel.

A comprehensive guide to data collection with definitions and rationale will be provided together with a paper version of the case report form (CRF). A comprehensive guide to accessing the data entry forms on the website and entering all follow-up data will be provided in the Data Completion Manual and Operations Manual. All of these documents will also be available in PDF format as required to assist the site research staff to ensure high-quality data collection and data entry.

11.1. Record retention

All paper study records, including consent documentation, paper CRFs (if used) and electronic records will be kept following the completion of the study: 15 years and otherwise as per local regulations in other jurisdictions.

12. Quality control and quality assurance monitoring

12.1. Responsibilities of the investigator

The site Principal Investigator agrees to perform the clinical trial in accordance with this clinical trial protocol, ICH guideline for Good Clinical Practice and all applicable regulatory requirements. The site Principal Investigator is required to ensure compliance with all procedures required by the clinical trial protocol and with all study procedures provided by the central or regional coordinating centre.

The site Principal Investigator agrees to provide reliable data and all information requested by the clinical trial protocol in an accurate, legible and timely manner according to instructions provided.

12.2. Responsibilities of the Coordinating centre

The central coordinating centre, The George Institute for Global Health - India, is responsible for taking all reasonable steps to ensure the proper conduct of the clinical trial protocol. The coordinating centre has multiple measures in place for data quality control. These measures include:

i. on-site training of research and clinical personnel
ii. standard operating procedures to guide storage and administration of the study drug
iii. ongoing assessment of quality metrics
iv. ongoing review of missing data and outliers
v. availability of Coordinating Centre personnel and the Principal Investigators 24/7 to answer study-related questions

12.2.1. Site Initiation

Prior to initiation of the study at each participating site, the central or regional coordinating centre will be responsible for providing adequate training to the site Principal Investigator and study personnel. The training will cover all aspects of the study protocol and procedures and will include practical training on the use of the web-based randomisation system, electronic CRF website and study materials. The site initiation visit will be conducted by teleconference, videoconference or face-to-face meeting at the participating site. Written and electronic materials will be supplied for study staff and for the education of clinical ICU staff at each participating site.

12.2.2 Monitoring during the study

A study monitor from the central or regional coordinating centre will ensure that the study is conducted according to the protocol, Good Clinical Practice guidelines and relevant regional regulatory requirements. The main duty of the study monitor is to help the investigator and the coordinating centre maintain a high level of ethical, scientific, technical and regulatory quality in all aspects of the trial.

The site Principal Investigator and study personnel will be available to discuss the study. These monitoring visits by phone or in person will include, but will not be limited to, review of the following aspects:

1. Adherence to the protocol including consistency with inclusion and exclusion criteria
2. HCW recruitment
3. Adverse event documentation and reporting
4. Compliance with the study assigned administration method
5. Compliance with regulations

The central coordinating centre team will conduct regular remote monitoring on the web-based database by applying validation and consistency rules and with regular data cleaning to ensure the integrity of the study data.

12.2.3. Site Close out

At completion of the trial, ensure secure facilities for the storage of study data as required by local regulations.

12.3. Management of protocol deviations

A protocol deviation is an unanticipated or unintentional departure from the expected conduct of an approved study that is not consistent with the current research protocol or consent document. A protocol deviation may be an omission, addition or change in any procedure described in the protocol.

The site investigator should not implement any deviation from or changes to the protocol without agreement by the study management committee and documented approval from the HREC / IRB of the amendment, except where necessary to eliminate an immediate hazard(s) to trial participants. In the event of an emergency intended to eliminate an apparent immediate hazard to participants the investigator may implement or omit any process as deemed appropriate.

Substantive deviations from the protocol must be documented and promptly reported to the study management committee and the HREC / IRB (if applicable). The report should summarise the event and action taken.

12.4. Access to data and documents

The study may be audited by government regulatory authorities, local HREC / IRBs or qualified representatives of The George Institute for Global Health as permitted by regulations. Therefore, access to other study related files, must be made available at all study sites for monitoring and audit purposes during the course of the study and after its completion.

Participants will be assigned a unique ‘participant study number’ and will not be identified by name in the study database. The site research staff will securely keep a list of participants and their corresponding study number, and confidentiality of information will be preserved. The confidentiality of the participant will be maintained unless disclosure is required by regulations.

13. Statistical methods

13.1. Sample size:

6,950 HCWs will be enrolled to detect a 25% relative reduction, or 2.5% absolute risk reduction, in the infection rate from an estimated baseline infection rate of 10%, with 80% power. This sample size allows for a potential loss to follow-up rate of 10% and a potential non-compliance rate of 10% in both the treatment and control arms.

Based on the premise that there are a minimum of 300-400 HCWs exposed to COVID-19 patients at each of the hospitals treating these patients, we will plan to recruit about 15-25 hospitals across the country. The investigators have strong collaborations with both public and private hospitals across the length and breadth of India and therefore achieving the sample size is feasible. As the study population will be drawn from a broad cohort of hospitals, the results are generalizable.

13.2. Statistical analysis plan

Categorical variables will be reported as numbers and percentages and continuous variables as mean/SD or median/IQR based on the distribution of data. For the primary comparison of symptomatic and confirmed COVID-19 disease between the groups, we will apply a chi-square test. We will develop a multivariable logistic regression model to adjust for differences in key baseline covariates (if any). For all secondary outcome measures, appropriate statistical tests will be employed.

A formal statistical analysis plan will be agreed upon and placed in the public domain before the study database is locked for the analysis of the primary outcome. The trial protocol will be written up for a formal publication in a peer reviewed journal. The trial will also be registered on the Indian clinical trials registry and on clinicaltrials.gov

13.3. Interim analysis

In order to address safety concerns, at least one formal interim analysis will be conducted at 90 days (where 25% of planned recruitment) has been completed and followed up for 4 weeks. The purpose of this interim analysis is to assess safety and efficacy according to a pre-specified DSMC Charter.

14. Publications and reports

The study will be conducted in the name of the 'HOPE Study Investigators'. Central project coordination and data management will be provided by The George Institute for Global Health, Delhi, India.

Authorship of publications arising from the study will be the management committee with full credit assigned to all collaborating Institutions, investigators and site research staff. Responsibility for the content of manuscripts will rest with the writing committee, and, where listed, the chair of the writing committee will be listed first with subsequent members listed alphabetically.

It is expected that findings will be disseminated via publication in high-quality peer reviewed journals in the medical literature. Study findings will also be presented at regional, national, international intensive care conferences, and results via social media and mainstream media platforms.

Funding bodies will be acknowledged in all publications.

14.1. Public Access

The protocol and statistical analysis plan will be made public prior to data analysis of the principal study. The participant level dataset will be made available at a time approved by the Management Committee.

15. References:

1. Available from: https://covid19.who.int/ (accessed on 1st May 2020)
2. Available from: https://www.who.int/dg/speeches/detail/who-director-general-s-opening-remarks-at-the-media-briefing-on-covid-19---11-march-2020 (accessed on 1st May 2020)
3. Available from: http://covidindiaupdates.in/ (accessed on 1st May 2020)
4. Wu Z, McGoogan JM. Characteristics of and Important lessons from the Coronavirus Disease 2019.JAMA 2020; doi:10.1001/jama.2020.2648
5. Guan W, Ni Z, Hu Y, Liang W, Ou C, He J et al. Clinical characteristics of Coronavirus Disease 2019 in China. N Eng J Med 2020 10.1056/NEJMoa2002032
6. Available from: https://www.cebm.net/covid-19/global-covid-19-case-fatality-rates/ (accessed on 27th March 2020).
7. Available from: https://www.epicentro.iss.it/coronavirus/sars-cov-2-sorveglianza-dati (accessed on 27th March 2020).
8. Available from: https://www.who.int/csr/sars/country/table2004_04_21/en/ (accessed on 27th March 2020).
9. Available from: http://applications.emro.who.int/docs/EMCSR246E.pdf?ua=1 (accessed on 27th March 2020).
10. Available from: https://economictimes.indiatimes.com/news/politics-and-nation/covid-19-outbreak-protective-health-gear-in-short-supply/articleshow/74765953.cms (accessed on 27th March 2020).
11. Available from: https://www.news18.com/news/india/coronavirus-cases-india-over-10-tested-for-covid-19-due-to-severe-respiratory-illness-found-positive-most-did-not-travel-overseas-2555317.html (accessed on 29th March 2020).
12. Available from https://www.cdc.gov/coronavirus/2019-ncov/hcp/therapeutic-options.html (accessed on 1st May 2020)
13. Liu J, Cao R, Xu M, Wang X, Zhang H, Hu H et al. Hydroxychloroquine, a less toxic derivative of chloroquine, is effective in inhibiting SARS-CoV-2 infection in vitro. Cell Discovery 2020;6(16) https://doi.org/10.1038/s41421-020-0156-0
14. Vincent MJ, Bergeron E, Benjannet S, Erickson BR, Rollin PE, Ksiazek TG et al. Chloroquine is a potent inhibitor of SARS coronavirus infection and spread. Virology Journal 2005;2(69) https://doi.org/10.1186/1743-422X-2-69
15. Available from: https://www.sciencemag.org/news/2020/03/who-launches-global-megatrial-four-most-promising-coronavirus-treatments (accessed on 27th March 2020).
16. Available from: https://edition.cnn.com/2020/03/23/africa/chloroquine-trump-nigeria-intl/index.html; https://www.livescience.com/coronavirus-chloroquine-self-medication-kills-man.html (accessed on 27th March 2020).
17. New Ethicals, 7th Edition.2001, Publisher Adis International. Plaquenil:Hydroxychloroquine sulphate, page 1486-88
18. Medsafe. New Zealand Date Sheet Plaquenil-hydroxychloroquine sulphate. https://www.medsafe.govt.nz/Profs/Datasheet/p/Plaqueniltab.pdf (accessed on 27th March 2020).
19. World Medical Association. Declaration of Helsinki - ethical principles for medical research involving human subjects. 2013.
20. National Health and Medical Research Council, The Australian Research Council, & The Australian Vice-Chancellor's Committee. National statement on ethical conduct in human research. Commonwealth of Australia, Canberra 2007 (updated 2015).
21. Available from: http://ethics.ncdirindia.org//asset/pdf/EC_Guidance_COVID19.pdf
