# Supplementary material for: Hydroxychloroquine plus personal protective equipment versus standard personal protective equipment alone for the prevention of COVID-19 infections among frontline healthcare workers: the HydrOxychloroquine Prophylaxis Evaluation(HOPE) trial: A structured summary of a study protocol for a randomized controlled trial
Source: Trials. 2020 Aug 31;21:754. doi: 10.1186/s13063-020-04679-3 (PMC7456761; doi:10.1186/s13063-020-04679-3)
Supplement: Supplementary file 2 — Additional file 2. [file 13063_2020_4679_MOESM2_ESM.pdf]

23 Jun 2020

To
Dr. Bharath Kumar,
Principal Investigator,
Apollo Hospitals,
Chennai, Tamil Nadu, India

Ref: IEC Application No: AMH-005/05-20

Protocol No: TGI-IN4673

Title: A randomized controlled trial of hydroxychloroquine (HCQ) as compared to standard care for the prevention of COVID-19 infections among healthcare workers exposed to SARS-CoV2

Sub: Final Full Board (Subsequent to your letter dated 05th & 17th June 2020).

Dear Dr. Bharath Kumar,

The Institutional Ethics Committee – Clinical Studies, Apollo Hospitals reviewed and discussed the documents submitted by you related to the conduct of above mentioned study at the meeting held on 22nd Jun 2020.

S.No	Contents	Version and Date
1.	Study Protocol	Version 3.0 dated 03-Jun-2020
2.	Protocol summary of changes	Version 3.0 dated 03-Jun-2020
3.	Participant Information Sheet & Consent Form-English	Version 1.0 dated 04-Jun-2020
4.	Hydroxychloroquine-Dug Information	dated 17 March 2020
5.	George Institute for Global Health-Approval letter	dated 5th May 2020
6.	CRF	Version 1.0 dated 03-Jun-2020
7.	Draft Clinical Trial Agreement	Not Applicable
8.	Fully Executed Clinical Trial Agreement	Dated 17th Jun 2020
9.	Participant Information Sheet & Consent Form Telugu	Version 1.0 dated 4th Jun 2020
10.	Participant Information Sheet & Consent Form Hindi	Version 1.0 dated 4th Jun 2020
11.	Participant Information Sheet & Consent Form Tamil	Version 1.0 dated 4th Jun 2020

The following Institutional Ethics Committee – Clinical Studies members were present at the meeting held on 22nd Jun 2020 at 03:00 pm via virtual meeting.

S. No.	Name	M/F	Qualification	Designation	Affiliated Y/N	Position in the committee
1.	Dr. R. Ramakrishnan	M	M.Sc., Ph.D, M.A.E	Consultant/ Ex -Scientist "G"	No	Chairperson

S. No.	Name	M/ F	Qualification	Designation	Affiliated Y/N	Position in the committee
2.	Dr. Rema Menon	F	MBBS	HOD (Blood Bank Transfusion Services)	Yes	Member Secretary
3.	Dr. Nirumal Rakkesh **	M	MBBS, M.D. (Pharmacology)	Pharmacologist	Yes	Alternate Basic Medical Scientist
4.	Dr. Arun Chander Yadav. K *	M	MBBS, M.D. (Pharmacology)	Pharmacologist	Yes	Basic Medical Scientist
5.	Dr. Rathna Devi	F	MBBS, DMRT	Sr. Consultant Radiation Oncologist	Yes	Member (Clinician)
6.	Dr. K. Sathyamurthi	M	M.A. (Social Work), PGDHRM, Ph.D	HOD Department of Research Programme in Social Work	No	Social Scientist
7.	Mr. Cibi Vishnu	M	B. Com., L.L.B., English Law	Lawyer	No	Lawyer
8.	Mrs. Padma Priya	F	B.A. (History) & M.A. (Hindi)	Home Maker	No	Layperson

*** Alternate Basic Medical Scientist*
** Dr. Arun Chander Yadav. K cited conflict of interest and didn't participate in the decision-making process.*

After due ethical and scientific consideration, the Ethics Committee has approved all the documents and the study to be conducted by you in the presented form.

Please note that the date of initiation of the study, the date of first patient and last patient participation and the site close out should be informed to the Ethics Committee. The Ethics Committee should also be informed about the progress of the study on **Half yearly basis.** Any changes in the protocol and patient information / informed consent, and a copy of the final clinical study report should be submitted Please submit a complaints and non compliance form to EC after each monitoring/inspection.. The AEs are to be reported before each EC meeting. Submit a report of protocol deviations/violations and serious adverse event as per regulatory timeline and mention the reason for delay, if any.

Please note the period of validity of this approval is for one calendar year and ends on 22nd Jun 2021.

The Institutional Ethics Committee – Clinical Studies is constituted and works as per ICH-GCP, National Ethical Guidelines for Biomedical and Health Research involving Human Participants (ICMR 2017) and New drugs and Clinical Trial Rules, March 2019.

Yours Sincerely,

MEMBER SECRETARY
INSTITUTIONAL ETHICS COMMITTEE CLINICAL STUDIES
APOLLO HOSPITALS, AHEL
CHENNAI, TAMIL NA[illegible]

Dr. RemaMenon, 24/06/2020
Member Secretary
Institutional Ethics Committee – Clinical Studies
Apollo Hospitals, Chennai
Tamil Nadu, India.

Status: Final Full BoardApproval

IEC Application No.:AMH-005/05-20(NOTE: Please quote this application no. in all your future communications)
